# Experiences in Formulating Insect-Based Feeds: Selected Physicochemical Properties of Dog Food Containing Yellow Mealworm Meal

**DOI:** 10.3390/ani15142087

**Published:** 2025-07-15

**Authors:** Remigiusz Gałęcki, Bartosz Pszczółkowski, Łukasz Zielonka

**Affiliations:** 1 Department of Veterinary Prevention and Feed Hygiene, Faculty of Veterinary Medicine, University of Warmia and Mazury in Olsztyn, 10-719 Olsztyn, Poland; 2 Department of Material and Machine Technology, Faculty of Technical Sciences, University of Warmia and Mazury in Olsztyn, ul. Oczapowskiego 11, 10-719 Olsztyn, Poland

**Keywords:** edible insects, *Tenebrio molitor*, pet food, nutritional value, companion animals, veterinary diets, fatty acid profile, amino acid profile, mechanical properties

## Abstract

Yellow mealworm (*Tenebrio molitor*) meal is a promising sustainable protein for pet food, yet its effect on nutrient balance and granule texture is incompletely defined. Five dry dog food formulas containing 25%, 30%, 35%, 40%, and 45% of *T. molitor* meal were extruded and analyzed for proximate composition, fatty-acid and amino-acid profiles, and compressive mechanical properties. Crude-protein concentration remained stable, whereas fat and ash increased (*p* < 0.01) and carbohydrates decreased as the inclusion level rose. Tenebrio molitor meal enriches granules in oleic, linoleic, and α-linolenic acids, improving the ω-6:ω-3 ratio from 8.0 to 5.4. Essential amino acid levels were conserved, although lysine and methionine fell modestly (≤11%). Mechanical testing showed a linear decline in hardness (331 → 300 N) and stiffness (46 → 41 N mm^−1^), indicating softer, more deformable granules at higher inclusion levels. The inclusion of up to 45% *T. molitor* meal can be incorporated without compromising protein quality while enhancing unsaturated fat content. Minor lysine/methionine dilution can be offset by targeted supplementation. These data support wider adoption of insect protein in hypoallergenic and eco-friendly canine diets.

## Simple Summary

Insects such as mealworms are increasingly explored as sustainable pet food ingredients with a high nutritional value and a smaller environmental footprint than traditional meat sources. This study evaluates the nutrient content and mechanical properties of five dog food formulas with different proportions of yellow mealworm meal. The formulas were analyzed for the content of protein, fat, fiber, and fatty acids, as well as mechanical resistance to deformation under load. It was found that higher mealworm content stabilized protein levels and increased the content of beneficial fats, such as omega-6 and omega-3 fatty acids, which are important for canine health. However, a slight decrease in the concentrations of some essential amino acids was noted with increasing insect meal levels, suggesting the need for careful diet formulation. The mechanical properties of the formulas also changed in response to higher mealworm content, resulting in softer and more flexible granules. These findings are important for developing sustainable, hypoallergenic dog foods that meet nutritional needs while supporting environmental goals.

## 1. Introduction

The edible insect sector is experiencing rapid growth due to its scalability, sustainability, and the increasing recognition of its potential to transform the global feed chain. Edible insects are rich in protein, fats, and micronutrients, and they are a viable alternative to conventional meat sources. Insect production requires significantly fewer resources, such as land and water, and has a lower environmental impact in terms of greenhouse gas emissions [1,2]. The European Union’s (EU) Novel Food Regulation [3] has established a legal framework for supporting the commercial development of the edible insect industry. This regulation ensures that insects introduced onto the EU feed market meet stringent safety standards [4]. Several insect species have already been approved for human consumption, including crickets (*Acheta domesticus*), yellow mealworms (*Tenebrio molitor*), and grasshoppers (*Locusta migratoria*) [5]. This legal endorsement has enabled the expansion of insect farming across Europe, leading to increased research and development projects to scale up production [6]. Despite regulatory progress, challenges remain, particularly with regard to consumer, including animal owners’ acceptance and the establishment of robust supply chains of insect meal for constant food and feed production [7,8,9]. Nevertheless, the edible insect sector continues to flourish, and it could play a pivotal role in addressing global food and feed security issues in the coming decades [10].

Edible insects also hold promise in animal nutrition, particularly in the production of feed for companion animals such as dogs and cats. Insects such as black soldier fly larvae (*Hermetia illucens*), crickets, and mealworms have been found to provide a high-protein, nutrient-dense alternative to conventional animal feeds [11,12]. Insects contain essential amino acids, fatty acids, and minerals that are critical for the health of companion animals [13]. Studies indicate that the nutritional value of insect-based pet food could be comparable to that of traditional protein sources, such as chicken or beef, while being more sustainable due to lower resource use and lower emissions in the production process [14]. The use of insects in pet food is still in its infancy, particularly on Western markets, where consumers may be hesitant to accept these novel protein sources [15]. However, the environmental benefits and the nutritional adequacy of insect-based pet food have prompted companies to explore this market, leading to the development of dog and cat foods incorporating insect meal [16]. As awareness grows and regulatory frameworks adapt, the use of edible insects in companion animal nutrition is expected to increase, potentially transforming the pet feed industry [17].

*Tenebrio molitor* is increasingly explored as a sustainable alternative protein source for pet food with an optimal nutritional profile. Mealworms are rich in high-quality protein, fat, and essential amino acids, which are crucial for the healthy growth of companion animals such as dogs and cats [18]. In terms of macronutrient levels, mealworms typically contain around 50% protein and 30% fat on a dry matter basis, and they are a rich source of fiber, which makes them an attractive feed ingredient [19]. *Tenebrio molitor* also contains essential vitamins (including vitamin B12) and minerals, such as calcium, zinc, and iron, which contribute to its overall nutritional value [20]. However, as a feed component, insect meal does not fully meet the amino acid requirements of dogs and cats. Insect meal offers a more environmentally friendly alternative to traditional protein sources such as beef or chicken, and research indicates that mealworms have a much smaller environmental footprint and require less water, land, and feed than conventional livestock [21]. Pet food producers could start to incorporate *T. molitor* meal into their current formulations as a key ingredient, thus providing nutritional balance and supporting sustainability in the pet food industry [22].

The use of *T. molitor* meal in animal feed is gaining traction, but its full potential has not been fully explored, especially in regard to the final nutritional outcomes for animals and the scalability of its integration. Mealworm meal is abundant in protein, essential fatty acids, and micronutrients, and it is a viable alternative to traditional protein sources such as fish meal and soy [23,24]. Moreover, *T. molitor* meal provides essential proteins and lipids, but it may be deficient in certain nutrients such as specific amino acids or vitamins, which implies that it has to be combined with other feed ingredients to fully meet the dietary needs of animals [25]. Meal processing methods (thermal euthanasia, hot-air drying, and fine milling) can influence nutrient quality [26]; excessive heat may reduce lysine availability or promote Maillard reactions. Moreover, large-scale production raises specific hazards, i.e., Salmonella spp. and *Bacillus* spp. spores and mycotoxin issues in European insect farms, emphasizing strict HACCP protocols [27]. In companion animal nutrition, insect meals are promising hypoallergenic proteins, but chitin level and processing must be optimized to preserve digestibility and safety [28].

The impact of *T. molitor* meal on the final product also depends on its interactions with other feed ingredients. Research has shown that *T. molitor* meal can be combined with plant-based and animal-based protein sources, such as soy and fish meal, respectively, to enhance the nutritional value of feed and create feeds that better meet the dietary needs of companion animals [29]. However, these combinations must be carefully formulated, as excessive reliance on a single ingredient can lead to nutrient imbalances or impact the feed’s palatability [22]. Attempts are being made to determine the optimal inclusion rates of mealworm meal in feeds to maximize its benefits without compromising the overall quality of the diet [21].

Several canine studies already suggest that *T. molitor* meal can replace part of the meat fraction without affecting nutrient digestibility or health. Bosch et al. [30] demonstrated that *T. molitor* protein delivers an essential amino acid score comparable with poultry by-product meal in dogs, while Feng et al. [31] showed that enzymatically hydrolyzed mealworm attractant, although initially less palatable than chicken-liver hydrolysate, still increased voluntary intake relative to an unflavored control. More recently, Lemos et al. [32] reported no adverse hematological or biochemical changes when adult dogs consumed extruded diets containing up to 7.5% mealworm meal for 14 days, and Premrov et al. [33] found that mite-allergic dogs exhibit only limited immunological cross-reactivity to *T. molitor* proteins.

Despite these encouraging data, the existing literature is restricted in two key respects. First, inclusion levels mostly remained below 10% of the diet’s dry matter; consequently, the technological and nutritional consequences of the higher *T. molitor* proportions (≥25%) needed to formulate truly meat-free, protein-adequate granules are unknown. Second, previous canine trials focused on digestibility, palatability, and short-term health markers but did not characterize how escalating *T. molitor* inclusion affects the physico-mechanical integrity of extruded granules—a critical quality attribute that adjusts breakage during transport, feed-intake behavior, and dental abrasion. As the industry moves toward more sustainable feed solutions, the role of *T. molitor* meal will likely increase, provided that technological and nutritional challenges are addressed through continued research and innovation [26,28].

The aim of this study is to evaluate the nutritional value of five dog food formulas with different inclusion levels of *T. molitor* meal (25%, 30%, 35%, 40%, and 45%). In addition, attempts were also made to assess the effect of varying dietary levels of mealworm meal on selected physical parameters of the formulas that influence their functionality in dogs’ diets.

## 2. Materials and Methods

### 2.1. Feed Production

Five feed formulas were developed. The formulas were characterized by varying inclusion levels of *T. molitor* meal at 25%, 30%, 35%, 40%, and 45% on a dry matter basis. Selected concentrations reflect realistic inclusion ranges currently considered in commercial and experimental insect-based dog foods and allow for observing linear and threshold effects on nutritional and mechanical outcomes. Live *T. molitor* larvae were sourced from a Polish insect farm considering HACCP feed safety requirements and national veterinary regulations concerning feed safety, animal health, disease control, animal welfare, and feed hygiene. To ensure the nutritional consistency of the purchased insects, larvae were sourced from the same supplier during the entire project. The mealworms were reared on a grain-based substrate (primarily wheat bran supplemented with vegetables for moisture), which is a standard diet for *T. molitor* production. Insects were transported and stored in line with ethical standards and IPIFF guidelines on insect welfare [34]. Before processing, insects were fasted for 72 h to clear the digestive tract. Mechanical cleaning methods were applied to remove feed residues, exuviae (molted skins), dead larvae, and pupating individuals. The larvae were euthanized using thermal methods (quick blanching at 90 °C) in line with AVMA guidelines [35], then dried and milled via a commercial insect-processing line. The resulting full-fat *T. molitor* meal was a dry, light brown color fine powder with approximately 8% moisture and the following nutrient profile: crude protein—52.3%, crude fat—31.8%, crude ash—4.6%, and crude fiber—5.1% (including chitin).

The nutritional composition of feed was designed to meet the following target parameters on a dry matter basis: crude protein content—approximately 28% (±2.5%); crude fat content—approximately 15% (±1.5%); crude ash content—approximately 5% (±2.5%); and crude fiber content—approximately 5% (±2.5%). The nutritional parameters were selected in accordance with the FEDIAF Nutritional Guidelines for Complete and Complementary Pet Food for Cats and Dogs [36], which provide standardized recommendations for the nutritional composition of pet foods. Depending on the formula, *T. molitor* meal was partially substituted with thermally processed potato by-products and fish oil. Specifically, the inclusion of thermally processed potato by-products (potato protein, starch, and potato peels) ranged from approximately 30% to 50% of the diet (dry matter basis) across the five formulas, while fish oil constituted about 0.5% to 6%. The potato by-products served as the main carbohydrate source and protein supplementation. Fish oil was the added fat source providing fatty acids. Importantly, the potato-by-products to fish-oil ratio was not uniform across the diets; each formula was individually adjusted to meet the target crude protein and crude fat levels. Thus, formulas with higher *T. molitor* meal inclusion required proportionally less potato by-products (especially potato protein) and fish oil, whereas those with lower insect meal used more potato by-product and oil to reach the nutritional targets. All other ingredients (e.g., vitamin, mineral premix, herbs, berries, and plantain) were kept constant across diets and were selected in accordance with common hypoallergenic diet compositions. It is important that each experimental diet was formulated specifically for this study rather than being based on any standard commercial diet. A 50 kg batch of each feed formula was produced and tested. The details of some technological processes constitute the subcontractor’s intellectual property. According to the provided information, the production process involved feed extrusion methods that are commonly applied in the pet food industry. The feed production system relied on EN ISO 9001 [37] and HACCP quality management standards. The feed was produced in a dry form as oval granules with a diameter of 15 mm.

### 2.2. Nutritional Value Assessment

A primary sample of 0.5 kg was collected from each batch of feed using a sampling probe for nutritional analysis. Each primary sample was then divided into five laboratory samples (n = 25 for each formula). Before analysis, laboratory samples were ground and homogenized using a laboratory mill. To determine dry matter content, samples were dried at 105 °C to constant weight. The proximate chemical composition (total protein, crude fat, crude fiber, total ash, and total carbohydrates) of each laboratory sample was determined according to the standard methods recommended by AOAC [38]. Specifically, crude fat content was determined by the Soxhlet extraction method with diethyl ether as the solvent (PN-ISO 6492:2005) [39]. Total ash content was determined by incineration in a muffle furnace at 580 °C for 8 h (PN-ISO 2171:1994) [40]. Total protein (N × 6.25) was determined by the Kjeldahl method (PN-EN ISO 5983-1:2005) [41]. Crude fiber content was determined according to PN-EN ISO 6865:2002 [42]. Total carbohydrate content was calculated using the following formula:

$$\begin{array}{l} \mathrm{Total}\;\mathrm{carbohydrates}\;(\%)\\ \;\;\;\;=100\%-[\mathrm{Moisture}\;(\%)+\mathrm{Crude}\;\mathrm{protein}\;(\%)\\ \;\;\;\;+\mathrm{Crude}\;\mathrm{fat}\;(\%)+\mathrm{Crude}\;\mathrm{fiber}\;(\%)+\mathrm{Total}\;\mathrm{ash}\;(\%)] \end{array}$$

The gross energy (GE) of each experimental diet was estimated from its proximate nutrient composition using standard caloric conversion factors for macronutrients. Specifically, GE (kcal per 100 g of diet, as-fed) was calculated as follows:

GE=(%Crude Protein×5.65 kcal/g)+(%Crude Fat×9.40 kcal/g)+(%Carbohydrate×4.15 kcal/g)

Metabolizable energy (ME) was then calculated using modified Atwater factors, which are appropriate for dog diets. The modified Atwater equation assigns 3.5 kcal/g for protein, 8.5 kcal/g for fat, and 3.5 kcal/g for carbohydrate. Thus, ME (kcal per 100 g) was computed as follows:

ME=(%Crude Protein×3.5 kcal/g)+(%Crude Fat×8.5 kcal/g)+(%Carbohydrate×3.5 kcal/g)

All energy values are expressed as kilocalories per 100 g of diet (as-fed).

### 2.3. Fatty Acid Profile

To determine the fatty acid profile of the formulas, lipids were extracted from the samples using the Folch procedure. Approximately 5 g of each sample was combined with 40 mL of a chloroform/methanol (2:1, *v*/*v*) mixture. The extract was filtered into a 100 mL flask, transferred to a glass tube, and evaporated at 60 °C under a nitrogen stream. The extracted lipids were stored overnight in a refrigerator. Next, the lipid residue was dissolved in 10 mL of a methylation mixture (chloroform/methanol/sulfuric acid, 100:100:1 *v*/*v*). A 1 mL aliquot of the obtained solution was transferred to a sealable glass ampoule. Fatty acid methylation was carried out in the ampoule at 100 °C for 2 h. The resulting fatty acid methyl esters (FAMEs) were analyzed by capillary gas chromatography with flame-ionization detection (FID) (PerkinElmer, Waltham, MA, USA). Individual fatty acids were identified by comparing their retention times to those of known standards (Supelco 37-component FAME Mix, Sigma-Aldrich, Saint Louis, MO, USA). Separation was performed on a ZB-FAME capillary column (30 m length; Sigma-Aldrich, Saint Louis, MO, USA). Each formula was analyzed in five replicates (n = 25).

### 2.4. Amino Acid Profile

To determine the content of amino acids, the samples were subjected to acid hydrolysis prior to chromatographic analysis. Approximately 10 mg of each homogenized sample was weighed into a glass ampoule, and 0.5 mL of a hydrolysis solution (6 M HCl containing 1% 2-mercaptoethanol and 3% phenol) was added. The ampoules were flame-sealed and heated in an oven at 110 °C for 24 h. After hydrolysis, the ampoules were cooled and then opened. The hydrolysates were placed in a vacuum dryer and dried at 60 °C under reduced pressure for 24 h to remove residual HCl. Dry residue was reconstituted in 1 mL of the buffer (10 mM ammonium formate in water, pH 3.2) and sonicated in an ultrasonic bath for around 15 s. The solution was then diluted 1:10 with acetonitrile, stirred, and centrifuged (14,000 rpm, 10 min). The supernatant was transferred to chromatography vials for analysis. The chromatographic analysis was performed using a Vanquish Core UHPLC system (Thermo Scientific, Waltham, MA, USA) coupled with a TSQ Fortis triple-quadrupole mass spectrometer (Thermo Scientific). Separation was achieved on a Kinetex HILIC column (150 × 2.1 mm, 2.6 µm; Phenomenex, Torrance, CA, USA) with mobile phase A (water/buffer 9:1) and mobile phase B (acetonitrile/buffer 9:1). Amino acids were identified by positive electrospray ionization (ESI) in selected reaction monitoring (SRM) mode. The parameters of parent and fragment ions of each amino acid are given in Table 1. Amino acids were quantified by preparing calibration curves for each amino acid standard. Each formula was analyzed in five replicates (n = 25).

### 2.5. Mechanical Analyses

Compressive strength tests were conducted using a TA.HD.Plus Texture Analyzer (Stable Microsystems, Surrey, UK). The textural parameters of feed granules were determined with a 35 mm cylindrical probe. The probe compresses the sample at a strain rate of 5 mm·min^−1^. The results were used to determine the following parameters; hardness was defined as the peak force (N) required to cause a fracture in the granule, which corresponds to the maximum point on the force–displacement curve. Fracturability was defined as the deformation (expressed as strain, %) at which the first fracture event occurred. It was calculated as the relative displacement of the probe at the point of first cracking by dividing the change in height of the granule (ΔL) by its initial height (L_0_) and expressing the result as a percentage as follows: strain (%) = |ΔL/L_0_| × 100%. Stiffness was determined from the initial slope of the linear section of the force–displacement curve (analogous to Young’s modulus) and calculated as the ratio of stress (σ) to strain (ε) in the elastic range. In practice, this was expressed as the tangent of the angle (α) between the linear area of the force–displacement curve and the displacement axis (OX). Thirty granules of each formula were used in compressive strength tests (n = 150).

### 2.6. Statistical Analysis

Prior to parametric testing, the assumptions of normality and homogeneity of variances were examined. Normality of residuals was evaluated with the Shapiro–Wilk test; homogeneity of variances was assessed with Levene’s test. Because both assumptions were met (*p* > 0.05), the data were analyzed with a one-way analysis of variance (ANOVA) using the inclusion level of *T. molitor* meal (25%, 30%, 35%, 40%, 45%) as the independent factor. When the ANOVA indicated a significant effect, Tukey’s honestly significant difference (HSD) test was applied for pairwise comparisons. Statistical significance was accepted at *p* < 0.05. Nutritional and fatty-acid and amino acid variables were determined in quintuplicate (n = 5 per diet), while mechanical parameters were obtained from 30 granules per formula (n = 30). All calculations were performed in Statistica 13.3 (TIBCO Software Inc., Palo Alto, CA, USA).

## 3. Results

### 3.1. Nutritional Value

The five experimental diets had very similar crude protein levels (around 25.7–25.9% on a dry matter basis), reflecting the intended formulation target. As expected, differences in protein content among formulas were minor and not statistically significant (*p* > 0.05). In contrast, crude fat levels showed a slight increasing trend with higher insect meal inclusion (from 13.6% in the Formula 25% to 14.1% in the Formula 45%). This rise in fat content was accompanied by a corresponding decrease in carbohydrate (nitrogen-free extract) content from approximately 43% to 42% as *T. molitor* meal replaced starchy ingredients. Statistical analysis confirmed that the changes in fat and carbohydrate were significant (*p* < 0.05) when comparing the lowest and highest inclusion levels, whereas protein remained unchanged (*p* > 0.05). Crude fiber and ash contents also increased modestly with insect meal inclusion (fiber from 5.16% to 5.29%; ash from 5.36% to 5.73%), consistent with the higher mineral and chitin content of *T. molitor* meal. Overall, partial replacement of conventional ingredients with mealworm meal did not significantly affect protein content but did significantly influence the fat, carbohydrate, and fiber fractions of the diets. Detailed data are presented in Table 2. The calculated GE and ME of the five diets were consistent, confirming that the macronutrient changes kept the caloric density virtually constant across all formulations. GE values ranged within a narrow range from 452.14 kcal/100 g in the 25% *T. molitor* meal diet to 453.58 kcal/100 g in the 40% diet, with an overall mean of 452.9 ± 0.6 kcal/100 g. The corresponding ME values ranged from 356.48 to 357.93 kcal/100 g, with a mean of 357.0 ± 0.6 kcal/100 g. The largest difference between any two formulations was <1.5 kcal/100 g for both GE and ME.

### 3.2. Fatty Acid Profile

The examined formulas differed in the content of saturated fatty acids (SFAs), monounsaturated fatty acids (MUFAs), n-6 polyunsaturated fatty acids (n-6 PUFAs), and n-3 polyunsaturated fatty acids (n-3 PUFAs).

ΣSFA rose from 7.77 ± 0.30 mg g^−1^ DM in the 25% diet to 13.45 ± 0.44 mg g^−1^ in the 45% diet; Tukey’s HSD showed that the 40% and 45% diets were significantly higher than the 25% and 30% diets (*p* < 0.05), while the 35% diet was intermediate.

ΣMUFA followed the same pattern, increasing from 11.26 ± 0.50 to 20.58 ± 0.60 mg g^−1^ (*p* < 0.01). Pairwise comparisons indicated significant differences between the extreme inclusion levels (25% vs. 40% or 45%; *p* < 0.05).

Polyunsaturated fractions also responded to insect inclusion: n-6 PUFA rose from 9.16 ± 0.51 to 14.97 ± 0.45 mg g^−1^, and n-3 PUFA from 1.15 ± 0.10 to 2.76 ± 0.16 mg g^−1^. In each case the 45% diet differed significantly from the 25% and 30% diets (*p* < 0.05).

These results confirm that increasing the proportion of *T. molitor* meal significantly elevates the levels of SFA, MUFA, and both PUFA classes in the finished feed. Detailed data are presented in Table 3.

One-way ANOVA revealed a significant diet effect for most quantified fatty acids (*p* < 0.05). The mean concentration of myristoleic acid (C14:1 cis-9) increased from 1.39 ± 0.32 mg/g in the 25% diet to 2.38 ± 0.38 mg/g in the 45% diet; values for the 40% and 45% diets were both higher than those for the 25% and 30% diets (*p* < 0.05). Palmitic acid (C16:0) more than doubled (6.45 ± 0.79 → 11.48 ± 0.13 mg/g), with the 40% and 45% diets differing significantly from the 25% and 30% diets (*p* < 0.05). Oleic acid (C18:1 cis-9) showed a similar pattern, rising from 8.69 ± 0.90 to 16.53 ± 0.20 mg/g; all inclusion levels ≥35% differed from the 25% diet (*p* < 0.01). Linoleic acid (C18:2 n-6) increased from 9.16 ± 0.98 to 14.97 ± 0.11 mg/g, and the 45% diet was significantly higher than the 25% and 30% diets (*p* < 0.01). In contrast, palmitoleic acid (C16:1 cis-9) varied only slightly (1.1–1.7 mg/g) and was not different among diets (*p* > 0.05), while α-linolenic acid (C18:3 n-3), although numerically higher at 45%, also failed to reach significance (*p* > 0.05). Several minor fatty acids, including caproic acid (C6:0), caprylic acid (C8:0), capric acid (C10:0), undecanoic acid (C11:0), lauric acid (C12:0), tridecanoic acid (C13:0), pentadecanoic acid (C15:0), heptadecanoic acid (C17:0), and eicosanoic acid (C20:0), were below the limit of detection (<0.04 mg/g) in all formulas. Overall, these data confirm that increasing *T. molitor* meal significantly enriches the diet in palmitic, oleic, and linoleic acids while leaving other fatty acids largely unchanged. Detailed data are presented in Table 4.

### 3.3. Amino Acid Profile

The amino acid composition of the feeds was relatively similar at different *T. molitor* inclusion levels. The developed formulas were most abundant in alanine, aspartic acid, and glutamic acid, and each of these amino acids made a significant contribution to total protein content. The formulas with the highest inclusion levels of mealworm meal (40% and 45%) were most abundant in several amino acids, but the overall differences were not significant. For instance, alanine content ranged from 7.22% (of protein, *w*/*w*) in Formula 25% to 8.16% in Formula 45%. The content of lysine, an essential amino acid, decreased from 4.35% in Formula 25% to 3.88% in Formula 45%. Methionine content was relatively low in all formulas and also decreased slightly from 1.53% to 1.34% with increasing inclusion levels of *T. molitor* meal.

The statistical analysis revealed no significant differences (*p* > 0.05) in the content of individual amino acids across the compared formulas. This observation indicates that the inclusion level of *T. molitor* meal (25% to 45%) did not induce significant changes in the amino acid profile of the feeds. The differences in the content of selected amino acids (lysine and methionine) were too small to be deemed significant. The amino acid profile of the formulas is presented in Table 5.

### 3.4. Mechanical Properties

In Formula 25%, mean fracturability was determined at 7.04% (SD = 0.55); mean hardness (crushing force) was 330.56 N (SD = 3.33), and stiffness (elastic modulus-like behavior) reached 46.2 N/mm (SD = 2.1). In Formula 30%, fracturability increased to 7.58% (SD = 0.49), which indicates that these pellets were more susceptible to deformation before cracking. Hardness was slightly lower than in Formula 25% at 324.10 N (SD = 4.01), and stiffness was determined at 44.8 N/mm (SD = 1.9). In Formula 35%, fracturability increased to 8.12% (SD = 0.50); hardness was determined at 315.44 N (SD = 5.12), and stiffness decreased to 43.5 N/mm (SD = 2.3). A further decline in mechanical properties was noted in Formula 40%, where fracturability reached 8.65% (SD = 0.60) and hardness—309.30 N (SD = 4.77). Formula 45% was characterized by the highest deformability, fracturability of 9.20% (SD = 0.58), and the lowest hardness at 300.22 N (SD = 5.50). Formula 45% was also characterized by the lowest stiffness at around 41.0 N/mm (SD = 2.5).

**Figure 1:**
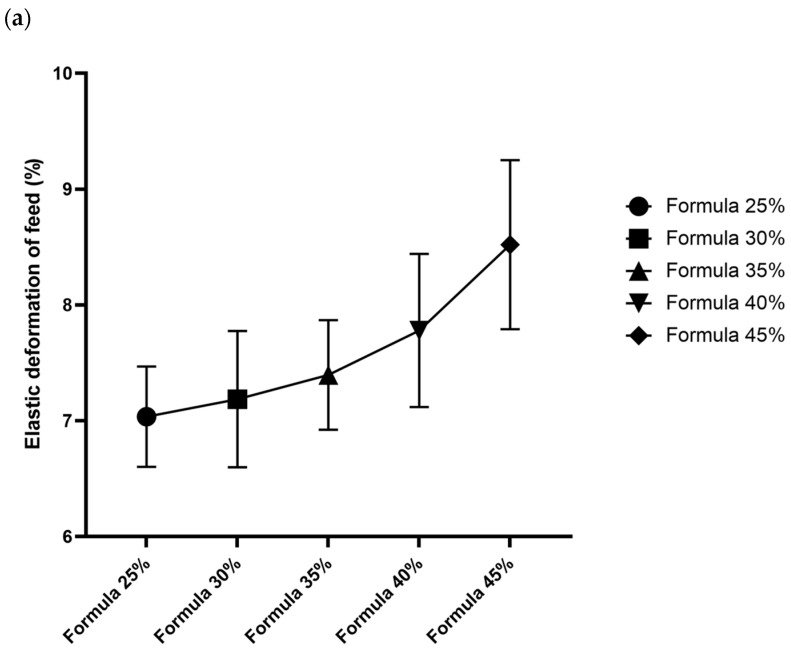

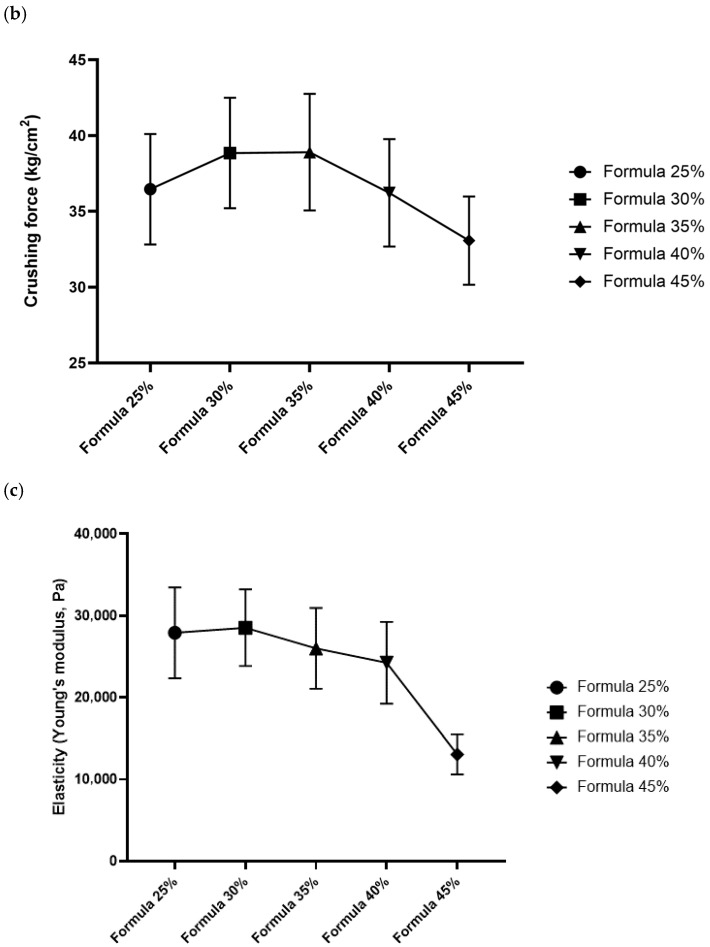
Mechanical properties of granules in five dog food formulas with increasing levels of *Tenebrio molitor* meal: (**a**) fracturability (% strain at first crack), (**b**) hardness (crushing force, kg/cm^2^), and (**c**) stiffness (resistance to deformation). Key: error bars represent SD (n = 30 per formula).

Feeds with higher inclusion levels of *T. molitor* meal were composed of granules that were generally softer (required less force to break) and more deformable (higher ability to tolerate strain before fracture). Stiffness tended to decline, which suggests that the granules of formulas with higher insect meal content were more elastic or less rigid. All differences in the mechanical parameters of the compared formulas were statistically significant (*p* < 0.05) in ANOVA and Tukey’s post hoc test. The post hoc test revealed that formulas with higher insect meal content (40%, 45%) were characterized by significantly higher elastic deformation and lower hardness than those with lower insect meal content (25%, 30%). Detailed data are presented in Figure 1.

## 4. Discussion

### 4.1. Nutritional Value

The study demonstrated that crude protein content was fairly similar in all formulas, whereas the content of crude fat, crude ash, and crude fiber increased gradually with a rise in the inclusion levels of mealworm meal. In contrast, carbohydrate concentration decreased as the levels of *T. molitor* meal increased, suggesting a shift in the macronutrient composition of the formulas. The detailed analyses of the nutritional profiles of each formula revealed minor variations in nutrient content, and the results may inform dietary formulations based on specific nutritional goals. The protein content of the studied formulas remained relatively stable, ranging from 25.73% to 25.9%. This observation suggests that the replacement of insect meal with other ingredients (potato by-products or fish oil) did not significantly affect crude protein content. Each diet was adjusted to approximately 28% crude protein by modulating the proportions of *T. molitor* meal and complementary ingredients—namely potato by-product (which contributes predominately to starch or potato protein) and added oil—so that the overall protein target was maintained. These results show that insect protein can be incorporated at varying inclusion levels without altering total dietary protein, provided the remaining ingredients are balanced appropriately. However, a slight increase in fat content was noted with a rise in the inclusion levels of insect meal, from 13.56% in Formula 25% to 14.07% in Formula 45%. These results corroborate the findings of other authors. Rumpold and Schlüter [43] conducted a comprehensive review of the nutritional value of edible insects, highlighting that *T. molitor* contains approximately 47–60% crude protein and 30–35% fat on a dry matter basis. Although the above values are higher than those observed in the current study, it should be noted that the analyzed formulas were designed to meet a specific crude protein target (28% ± 2.5%), which implies that insect meal was likely diluted with other ingredients to achieve this target. Comparable nutrient ranges were obtained by Penazzi et al. [44], who produced extruded dog granules in which *Hermetia illucens* larva meal replaced poultry meal at inclusion levels of 10% and 20% of the total diet. Their finished products contained 24–26% crude protein and 11–15% crude fat, values that closely mirror the protein (≈26%) and fat (≈14%) levels recorded here. Penazzi et al. [44] further showed that the insect-based diets matched control diets for palatability and digestibility, underscoring the practicality of using insect meals as primary protein sources. Moreover, Bosch et al. [30] analyzed various insect species, including *T. molitor*, and confirmed that insects offer a macronutrient profile that can be suitable for dog food, particularly in terms of protein and fat content. The cited authors also concluded that insects can be a rich source of selected essential amino acids, which is a crucial consideration in the process of formulating complete diets for dogs.

### 4.2. Fatty Acid Profile

The content of SFAs, MUFAs, and PUFAs (n-6 and n-3) increased with a rise in the inclusion levels of *T. molitor* meal. This indicates that insect meal can be a valuable source of fatty acids for dog food formulas. It should be noted that fish oil content, which varied slightly between diets, also influenced the fatty acid profiles. Formulas with higher fish oil content provided relatively higher amounts of omega-3 fatty acids, whereas formulas with lower fish oil content relied more on natural unsaturated fatty acids derived from mealworm meal (e.g., α-linolenic acid). Consequently, variation in fish oil content contributed to some of the observed differences in n-3 polyunsaturated fatty acid levels across diets. This observation may have important implications for the nutritional value and health benefits of these formulas. Mealworm meal was abundant in several saturated and unsaturated fatty acids, including palmitic, stearic, oleic, linoleic, and alpha-linolenic acids, whereas many other fatty acids were below the limit of detection.

The designed formulas differed significantly in fatty acid composition, and the concentrations of both SFAs and MUFAs increased with a rise in the inclusion levels of insect meal. Notably, oleic acid (C18:1) content doubled from 8.69 mg/g in Formula 25% to 16.53 mg/g in Formula 45%, while the content of linoleic acid (C18:2), an essential n-6 PUFA, also increased significantly from 9.16 mg/g to 14.97 mg/g.

Similar observations were made by Oonincx et al. [45], who studied the lipid composition of various edible insects, including *T. molitor*. Their research highlighted that *T. molitor* is a rich source of oleic and linoleic acids that are essential for maintaining healthy skin and coat in dogs. Borrelli et al. [46] also found that the content of n-6 and n-3 PUFAs increased with a rise in the inclusion levels of insect meal and concluded that insect meal can be an excellent source of unsaturated fatty acids, especially in comparison with conventional meat-based ingredients.

These findings are important because dogs require specific ratios of omega-6 to omega-3 fatty acids to maintain optimal health. Omega-3 and omega-6 PUFAs provide health benefits by modulating the immune response and exerting anti-inflammatory effects, and they can be particularly helpful in managing conditions such as osteoarthritis [47] and atopic dermatitis [48]. Diets rich in n-3 fatty acids can improve renal function by promoting balanced inflammatory responses and supporting cardiovascular health, which is especially important for older or at-risk dogs [49,50]. In the current study, the concentrations of n-3 and n-6 PUFAs increased steadily with a rise in the inclusion levels of mealworm meal (from 1.15 mg/g to 2.76 mg/g and from 9.16 mg/g to 14.97 mg/g, respectively), which aligns with the growing body of evidence that the synergistic effects of these fatty acids enhance overall canine health. Furthermore, the proportion of MUFAs such as C18:1 cis 9 is also significantly higher in formulations with higher fat content, which may improve energy metabolism and further benefit active or aging dogs [51]. The present results indicate that feeds with a higher content of insect meal may have a more balanced fatty acid profile, which may be particularly beneficial for dogs with inflammatory conditions or skin sensitivities. Some of the detected fatty acids also have antibacterial properties [52].

### 4.3. Amino Acid Profile

The study reveals slight variations in the amino acid content of the analyzed formulas. The concentration of some amino acids was fairly stable, whereas greater fluctuations were noted in the levels of other amino acids. Formulas with the highest inclusion levels of *T. molitor* meal were most abundant in several amino acids (including alanine, aspartic acid, and glutamic acid), which implies that insect meal could enhance their nutritional value.

Minor differences were observed in the amino acid profile. Concentrations of most amino acids did not vary significantly across diets containing 25–45% mealworm meal, indicating that the amino acid contribution of the insect ingredient was comparable to that of the proteins it replaced. Nevertheless, the observed differences were small and statistically non-significant (*p* > 0.05), showing that the combination of *T. molitor* meal with potato by-product and the other diet components produced a balanced amino acid profile. Uniform processing conditions (e.g., extrusion temperature) may also have resulted in similar amino acid retention across all diets, thereby preserving relative profiles. The studied formulas were most abundant in alanine, aspartic acid, and glutamic acid, while a slight decrease in lysine content was noted as the proportion of *T. molitor* meal increased. Similar results were reported by Gasco et al. [53], who evaluated the amino acid content of insect-based dog food and found that *T. molitor* meal can provide adequate levels of essential amino acids for dogs. However, the fact that higher inclusion levels of mealworm meal induced a slight decrease in lysine content could be of concern because lysine is an essential amino acid for dogs that promotes muscle development and overall health. Ravzanaadii et al. [20] found that while insect-based proteins are generally rich in essential amino acids, lysine content is sometimes lower compared to conventional meat proteins, which implies that commercial formulations may require supplementation to meet dietary requirements. Despite these concerns, the present study demonstrated that the amino acid profile of insect-based meals, in particular *T. molitor* meal, is comparable to that of traditional meat proteins. The presence of other essential amino acids, such as methionine and cystine, in adequate quantities supports the nutritional viability of these formulas. While edible insects are rich in many nutrients, they tend to be relatively low in methionine compared to traditional sources of animal protein. Methionine is an essential amino acid for dogs because it plays a key role in keratin production and overall protein metabolism, and diets deficient in this amino acid may lead to poor coat quality, hair loss, dullness, and skin problems. Therefore, diets based heavily on insect protein may require methionine supplementation to ensure optimal health and coat condition in dogs.

It should be noted that tryptophan was not included in the amino acid analysis due to its degradation during acid hydrolysis, which was the method employed in this study. Accurate quantification of tryptophan requires separate alkaline hydrolysis and specialized detection methods. Future studies should incorporate appropriate analytical protocols to assess tryptophan levels, given their essential role in canine nutrition.

### 4.4. Mechanical Properties

Mechanical tests revealed that higher inclusion levels of *T. molitor* meal increased the softness and flexibility of feed granules. Specifically, formulas containing higher proportions of insect meal were characterized by higher elastic deformation (granules could be compressed further before breaking) and lower breaking strength. In practical terms, formulas with higher inclusion levels of *T. molitor* meal consisted of granules that were easier to chew (softer) and less brittle (more flexible). The above could affect the palatability and chewiness of dog food. For instance, very hard granules might provide dental benefits by removing plaque, but they could be difficult to chew for puppies or older dogs with dental issues. These groups of animals require dry food with slightly softer granules.

The mechanical changes observed in studied granules might be influenced by processing method and the nature of the *T. molitor* ingredient. Studied feeds were produced by extrusion, whereas Purschke et al. [54] investigated the properties of *T. molitor* larvae under different processing conditions (pre-treatments like blanching and various drying methods) prior to incorporation into foods. Their study showed that processing can significantly affect the physico-chemical characteristics of insect meals—for instance, how proteins denature or how lipids are distributed. In this case, the *T. molitor* meal was thermally processed and dried in a way that retained much of its natural oil. When this full-fat insect meal was extruded into granules, the high lipid content likely acted as a plasticizer in the dough, leading to softer, less brittle granules. This reasoning is consistent with general feed technology knowledge: diets with higher fat tend to produce less dense, more pliable extrudates because fats lubricate and disrupt the starch-protein matrix. Additionally, *T. molitor* protein may differ from plant proteins in how it contributes to the granule structure. Purschke et al. [54] observed that the drying method of insects can alter protein functionality and fiber structure. The *T. molitor* meal in this study, produced via a standardized drying process, might have had lower water-binding capacity or a different particle structure compared to grain flours, resulting in granules that fracture more easily under stress. In simpler terms, replacing a portion of starchy binder with insect meal yielded a product that was mechanically softer. This explains why hardness dropped and elasticity increased as studies went from 25% to 45% insect inclusion.

Meléndez-Rodríguez [55] emphasized that mechanical resilience plays an important role in feed matrices by preserving their shape and integrity during handling and storage. Formulas with higher inclusion levels of insect meal were softer, but their structural integrity was preserved. Crumbling was not observed during normal handling, but these formulas were more likely to break under lower stress in mechanical tests. According to Recupido et al. [56], changes in the composition of bio-based composite materials could lead to increased softness at higher inclusion levels of certain components. By analogy, the present findings suggest a similar effect: the flexibility of granules increased with a rise in the inclusion levels of *T. molitor* meal. This could affect product durability, as softer granules might be more prone to breakage in transport. However, the hardness of all studied formulas was within the range of values typical of dry dog food.

In feeding scenarios, flexible granules could be more desirable for dogs with certain health issues (such as weaker jaws or dental concerns) and could potentially increase palatability due to ease of chewing. In the future, dog food manufacturers might consider combining insect meals with texturizing agents or adjusting processing conditions to fine-tune the hardness and brittleness of granules.

### 4.5. Compliance with FEDIAF Guidelines

The developed formulas were examined for fatty acid and amino acid content to assess their compatibility with the European Pet Food Industry Federation (FEDIAF) Nutritional Guidelines for Dogs [36] and ensure that they meet standard requirements for canine diets. According to FEDIAF, canine diets should have a balanced ratio of fatty acids (with sufficient levels of linoleic acid, an omega-6 fatty acid, and alpha-linolenic acid, an omega-3 fatty acid) and provide all essential amino acids above the minimum recommended amounts. The dietary fats for canine nutrition should include a balance of SFAs, MUFAs, and PUFAs, particularly omega-6 and omega-3 PUFAs, which are essential for skin health, coat quality, and inflammatory modulation. The SFA content of the tested formulas increased progressively with a rise in the inclusion levels of *T. molitor* meal, from 7.77 mg/g in Formula 25% to 13.45 mg/g in Formula 45%, which indicates that these feeds provide adequate amounts of energy and support cell membrane integrity. The content of MUFAs also increased from 11.26 to 20.58 mg/g, which is a desirable outcome because these fatty acids are a source of readily metabolizable energy, and their beneficial impact on cardiovascular health has been demonstrated in other species. Notably, the content of omega-6 PUFAs increased from 9.16 to 14.97 mg/g in the studied formulas, which is consistent with FEDIAF’s recommendations concerning skin health and immune function. In turn, the content of omega-3 PUFAs increased from 1.15 to 2.76 mg/g, indicating that the anti-inflammatory effects of the tested formulas are slightly below the ideal ratios and that additional supplementation may be required to achieve the optimal balance.

Based on FEDIAF’s guidelines, the formulated feeds were also analyzed for the content of essential amino acids that are crucial for muscle health, immune function, and enzyme activity in dogs [36]. The content of alanine, an important glucogenic amino acid, ranged from 7.22% to 8.16%, indicating that alanine levels were high enough to trigger gluconeogenic pathways that are necessary during endurance or stress. Arginine, which plays a key role in immune function and ammonia removal, was within the recommended levels in all formulas (4.49–4.79%). The formulas were also sufficiently abundant in histidine (2.00–2.29%) which is necessary for histamine synthesis and hemoglobin structure. However, lysine levels (4.35–3.88%) tended to decline in formulas with the highest inclusion levels of insect meal, which implies that they should be monitored to ensure adequate supply of lysine that plays a role in calcium absorption and bone health. Methionine levels also decreased from 1.53% to 1.34% and approximated the minimum recommended intake levels, which suggests that the designed formulations may need to be adjusted to enhance protein synthesis and antioxidant protection.

### 4.6. Practical Consideration and Study Limitations

While this study provides detailed insights into the chemical composition and physical properties of dog food formulas containing *T. molitor* meal, it did not assess the in vivo digestibility or bioavailability of nutrients in dogs. From a palatability standpoint, insects appear to be well-accepted by dogs. The inclusion of mealworm meal at up to 45% did not produce any off-putting odor or texture in the granules (as observed during production), and own feeding trials with insect-based diets suggests high acceptance in dogs.

Future studies should include digestibility trials in dogs to evaluate the true nutritional value and bioefficacy of *T. molitor* meal in companion animal diets. Such data would be essential for confirming the suitability of insect-based ingredients for long-term feeding and health outcomes in pets. Although the nutritional and mechanical properties of the developed dog food formulas were comprehensively evaluated in vitro, in vivo feeding trials have not yet been conducted. These tests are necessary to assess palatability, digestibility, and long-term health effects in dogs. Future research will focus on clinical validation of the developed diets in canine models, particularly dogs with food-responsive enteropathies, to verify their hypoallergenic potential and overall efficacy. The outcomes of such studies will provide critical insights into the practical application of insect-based feeds in companion animal nutrition.

The actual absorption and utilization of amino acids, fatty acids, and other nutrients from insect meal in the canine gastrointestinal tract remain critical aspects of nutritional efficacy. Insect-derived proteins are generally digestible; however, factors such as chitin content, processing methods, and matrix interactions may affect nutrient availability. Recent studies have shown that dogs fed diets where insect meal provided the sole protein source had protein digestibility coefficients comparable to conventional meat diets [57,58]. A similar outcome is expected for the studied formulations. One component unique to insects is chitin, the structural polysaccharide in their exoskeleton. Chitin is considered a form of dietary fiber since dogs lack enzymes to digest it. In our diets, the chitin contributed to the crude fiber content (~5% DM) and likely passed into the hindgut. While high chitin levels may slightly reduce protein digestibility by encapsulating some nutrients, the levels present here (mealworm meal contains roughly 5–10% chitin) are moderate. This amount of chitin may even have positive effects, such as stimulating beneficial gut microbiota (acting as a fermentable fiber) and improving stool quality. Nonetheless, if insect meal was used at higher inclusion or not finely milled, chitin could become a limiting factor for amino acid availability. Future diet formulations might consider enzyme additives (e.g., chitinase) or using insect protein concentrates (with reduced chitin) to further enhance nutrient assimilation. The fatty acids provided by the mealworm meal (and the supplemental fish oil) are not only beneficial but also digestible. Dog studies have indicated that insect-derived fats are well utilized, with high apparent fat digestibility [52]. The unsaturated fatty acids (like oleic, linoleic, and alpha-linolenic) abundant in studied high-insect diets should be readily absorbed and can confer anti-inflammatory and skin health benefits. It is advised to include antioxidants in formulas to protect these delicate fats from oxidation.

It is worth noting that a principal component analysis (PCA) could be a useful next step to analyze the fatty acid profile changes in a multivariate context. PCA would allow us to combine the numerous fatty acid variables into principal components, potentially revealing clear patterns or clusters among the different formulas. Such an analysis could confirm that the overall fatty acid profile shift is systematically associated with *T. molitor* inclusion level. Although we did not include a PCA here, this approach could provide an integrated view of the data beyond the single-factor comparisons and could be a valuable tool for future investigations.

## 5. Conclusions

*Tenebrio molitor* meal can be effectively incorporated into dog food formulas at inclusion levels of up to 45% without exerting a detrimental effect on the content of essential nutrients. The formulated diets were characterized by stable protein levels, and fat content and fatty acid quality improved with a rise in the inclusion levels of insect meal. A slight decrease in the concentrations of selected essential amino acids (lysine and methionine) suggests that insect meal could be combined with other protein sources or supplements to fully meet all essential amino acid requirements of dogs. The mechanical properties of dog food granules were influenced by insect meal, and formulas with higher inclusion levels of insect meal were characterized by softer and more deformable granules. This observation indicates that processing conditions or binders may need to be adjusted in large-scale production to maintain the integrity of granules in feeds with high proportions of insect meal.

Overall, the study demonstrated that higher inclusion levels of *T. molitor* meal significantly alter the nutritional composition and physical characteristics of dog food, but these changes are generally manageable and can be optimized. The developed insect-based formulas meet current nutritional guidelines for adult dogs and offer a promising solution for hypoallergenic diets because *T. molitor* is not a common allergen in pets. The formulas also contribute to sustainability goals by utilizing a protein source with a lower environmental footprint. These findings indicate that insect-based feeds are a sustainable and wholesome option in canine nutrition. Further research and development should focus on optimizing amino acid profiles (through complementary ingredients or supplementation), palatability testing in pets, and long-term feeding trials to ensure positive health outcomes. The growing use of *T. molitor* meal in pet food could play a part in reducing reliance on traditional livestock proteins, thereby supporting environmental sustainability while maintaining pet health.

## 6. Patents

The present study gave rise to patent P.443579—hypoallergenic dog food. The patent covers a hypoallergenic feed formulation for dogs suffering from food-responsive enteropathies. Hypoallergenic dog food contains *T. molitor* meal (inclusion level: 10% to 60%, preferably 35%), potato and/or sweet potato by-products (10% to 65%, preferably 48%), plant substrates of the plantain family (0.01% to 15%, preferably 7.3%), animal or vegetable fat (0.1–15%), herbs and minerals (0.01% to 5%, preferably 1.8%), berries (0.01% to 8%, preferably 0.6%), functional additives such as water- and fat-soluble vitamins (0.01% to 15%, preferably 6.3%), and plants of the flax family (0.01% to 5%, preferably 1%). The patented formulation has the following nutritional composition: crude protein—11% to 41%, (preferably 25%); crude fat—3% to 27%, (preferably 15%); crude ash—1% to 9%; (preferably 5%); and crude fiber—1% to 9%, (preferably 5%).

## Figures and Tables

**Table 1:** Parameters of parent and fragment ions in the chromatographic analysis of amino acids.

Amino Acid	Parent Ion (*m*/*z*)	Fragment Ion (*m*/*z*)
Alanine	90.014	43.970
Arginine	175.100	70.054
Cystine	241.024	151.970
Phenylalanine	166.062	120.000
Glycine	75.988	30.196
Histidine	156.074	110.054
Hydroxyproline	132.075	68.042
Aspartic acid	134.052	74.042
Glutamic acid	148.062	84.125
Leucine/Isoleucine	132.112	86.196
Lysine	147.112	84.125
Methionine	150.062	133.071
Proline	116.112	70.071
Serine	106.062	60.071
Threonine	120.088	103.125
Tyrosine	188.114	147.125
Valine	118.112	72.071

Key: *m*/*z*—mass-to-charge ratio.

**Table 2:** Mean content (%) of crude protein, fat, ash, fiber, and carbohydrates on a dry matter basis in five dog food formulas with increasing inclusion levels of *Tenebrio molitor* meal.

Formula	Crude Protein (%)	Crude Fat (%)	Crude Ash (%)	Crude Fiber (%)	Carbohydrates (%)
25%	25.77 ± 0.92	13.56 ± 0.78	5.36 ± 0.55	5.16 ± 0.59	43.15 ± 1.46
30%	25.73 ± 0.87	13.68 ± 0.68	5.41 ± 0.59	5.17 ± 0.65	43.01 ± 1.36
35%	25.83 ± 0.78	13.79 ± 0.65	5.37 ± 0.43	5.22 ± 0.49	42.79 ± 1.04
40%	25.75 ± 0.95	13.92 ± 0.68	5.42 ± 0.67	5.20 ± 0.57	42.71 ± 1.65
45%	25.90 ± 1.34	14.07 ± 0.86	5.73 ± 0.83	5.29 ± 0.69	42.01 ± 2.04

Key: the presented values are means ± SD (n = 5 per formula). Annotation: insect meal was replaced with potato by-products or fish oil in the same proportions to maintain the original parameters.

**Table 3:** Fatty acid profile of five dog food formulas with increasing inclusion levels of *Tenebrio molitor* meal. The table presents the concentrations (in mg/g feed, dry matter basis) of the following major fatty acid classes: saturated fatty acids (SFAs), monounsaturated fatty acids (MUFAs), omega-6 polyunsaturated fatty acids (n-6 PUFAs), and omega-3 polyunsaturated fatty acids (n-3 PUFAs).

Formula (% *T. molitor* Meal)	SFAs (mg/g)	MUFAs (mg/g)	n-6 PUFAs (mg/g)	n-3 PUFAs (mg/g)
25%	7.77 ± 0.30	11.26 ± 0.50	9.16 ± 0.51	1.15 ± 0.10
30%	8.90 ± 0.35	13.58 ± 0.62	10.74 ± 0.48	1.60 ± 0.12
35%	10.12 ± 0.41	16.02 ± 0.70	12.83 ± 0.53	2.10 ± 0.15
40%	11.30 ± 0.37	18.43 ± 0.55	13.64 ± 0.50	2.45 ± 0.14
45%	13.45 ± 0.44	20.58 ± 0.60	14.97 ± 0.45	2.76 ± 0.16

Key: the presented values are means ± SD (n = 5 per formula).

**Table 4:** Fatty acid profile of five dog food formulas with increasing inclusion levels of *Tenebrio molitor* meal. The table presents the concentrations (in mg/g feed, dry matter basis) of the following major fatty acids.

Fatty Acid	25%	30%	35%	40%	45%
C14:1 cis-9	1.39 ± 0.32	1.52 ± 0.28	1.95 ± 0.53	2.18 ± 0.33	2.38 ± 0.38
C16:0	6.45 ± 0.79	7.31 ± 0.18	8.92 ± 0.97	10.28 ± 0.22	11.48 ± 0.13
C16:1 cis-9	1.18 ± 0.13	1.17 ± 0.17	1.08 ± 0.39	1.65 ± 0.10	1.68 ± 0.21
C18:0	1.32 ± 0.22	1.30 ± 0.32	2.03 ± 0.52	2.09 ± 0.63	1.96 ± 0.04
C18:1 cis-9	8.69 ± 0.90	10.95 ± 0.44	13.15 ± 1.68	14.95 ± 1.42	16.53 ± 0.20
C18:2 cis-9,12	9.16 ± 0.98	9.91 ± 0.69	12.27 ± 0.54	12.92 ± 0.90	14.97 ± 0.11

Key: the presented values are means ± standard deviation (n = 5). In all formulas, the content of the following fatty acids was below the limit of detection (<0.04 mg/g) C6:0, C8:0, C10:0, C11:0, C12:0, C13:0, C14:0, C15:0, C15:1 cis-10, C17:0, C17:1 cis-10, C18:1 trans-9, C18:2 trans-9,12, both C18:3 isomers, C20:0, C20:1 cis-11, C20:2 cis-11,14, C21:0, the C20:3 isomers, C20:4 cis-5,8,11,14, C22:0, C22:1 cis-13, C20:5 cis-5,8,11,14,17, C22:2 cis-13,16, C23:0, C24:0, C24:1 cis-15, and C22:6 cis-4,7,10,13,16,19.

**Table 5:** Amino acid concentrations (% of total protein) in five dog food formulas with increasing inclusion levels of *Tenebrio molitor* meal.

Amino Acid	25%	30%	35%	40%	45%
Alanine	7.22 ± 0.16	8.04 ± 0.24	7.71 ± 0.07	7.56 ± 0.27	8.16 ± 0.03
Arginine	4.52 ± 0.36	4.79 ± 0.28	4.73 ± 0.33	4.71 ± 0.35	4.49 ± 0.18
Cystine	4.67 ± 0.14	5.61 ± 0.20	4.91 ± 0.33	5.12 ± 0.07	5.02 ± 0.18
Phenylalanine	6.07 ± 0.38	5.33 ± 0.17	5.51 ± 0.16	5.25 ± 0.43	5.41 ± 0.07
Glycine	5.71 ± 0.07	6.41 ± 0.58	5.84 ± 0.23	5.57 ± 0.39	5.94 ± 0.46
Histidine	2.00 ± 0.16	2.29 ± 0.14	2.11 ± 0.10	2.20 ± 0.17	2.16 ± 0.25
Aspartic acid	10.25 ± 0.58	9.92 ± 0.35	9.41 ± 0.92	10.01 ± 0.89	11.52 ± 0.02
Glutamic acid	16.88 ± 2.28	17.82 ± 0.65	18.52 ± 1.16	19.05 ± 0.78	17.97 ± 0.61
Leucine	8.03 ± 0.23	7.27 ± 0.41	7.63 ± 0.18	7.28 ± 0.54	7.34 ± 0.51
Lysine	4.35 ± 0.33	4.00 ± 0.06	4.05 ± 0.35	3.98 ± 0.15	3.88 ± 0.23
Methionine	1.53 ± 0.06	1.57 ± 0.10	1.46 ± 0.11	1.44 ± 0.11	1.34 ± 0.03
Proline	6.07 ± 0.50	5.89 ± 0.42	6.48 ± 0.13	6.35 ± 0.16	6.19 ± 0.42
Serine	4.55 ± 0.45	4.61 ± 0.06	4.30 ± 0.22	4.50 ± 0.21	4.50 ± 0.37
Threonine	4.41 ± 0.49	3.82 ± 0.05	3.94 ± 0.21	3.93 ± 0.11	3.89 ± 0.18
Tyrosine	5.87 ± 0.30	5.29 ± 0.28	5.48 ± 0.18	5.20 ± 0.24	4.50 ± 0.23
Valine	7.87 ± 0.54	7.36 ± 0.70	7.93 ± 0.01	7.84 ± 0.14	7.69 ± 0.47

Key: the presented values are means ± SD (n = 5 per formula).

## Data Availability

Data are presented in the article.
